# Growth Differentiation Factor 15 and Risk of Death in Haemodialysis Patients

**DOI:** 10.1155/2023/5163548

**Published:** 2023-03-23

**Authors:** Christelle Calen, Seraina Von Moos, Pietro Cippà, Alexandre Mebazaa, Mattia Arrigo

**Affiliations:** ^1^Division of General Internal Medicine, Hirslanden Zurich, Zurich, Switzerland; ^2^Department of Nephrology, University Hospital Zurich, Zurich, Switzerland; ^3^Division of Nephrology, Ente Ospedaliero Cantonale, Ospedale Regionale di Lugano, Lugano, Switzerland; ^4^Department of Critical Care and Anesthesiology, Lariboisiere University Hospital, and University of Paris, Paris, France; ^5^Department of Internal Medicine, Stadtspital Zurich Triemli, Zurich, Switzerland

## Abstract

**Aim:**

Noninvasive identification of haemodialysis patients at high risk of cardiovascular events and death might improve their outcome. Growth differentiation factor 15 is a prognostic biomarker in multiple disease entities, including cardiovascular disease. The aim of this study was to assess the association between plasma GDF-15 and mortality in a cohort of haemodialysis patients.

**Methods:**

Circulating GDF-15 was measured in 30 patients after a regular haemodialysis session, followed by a clinical follow-up for all-cause death. Measurements were performed using the Proseek Multiplex Cardiovascular disease panels (Olink Proteomics AB) and validated using the Elecsys GDF-15 electrochemiluminescence immunoassay on a Cobas E801 analyzer (Roche Diagnostics).

**Results:**

During a median of 38 months, 9 patients (30%) died. Seven deaths occurred in the group of patients with a circulating GDF-15 above the median and two in the group with lower GDF-15. Mortality was significantly higher in patients with circulating GDF-15 levels above the median, log-rank*P* = 0.044. The performance of circulating GDF-15 to predict long-term mortality has an area under the ROC curve of 0.76, *P* = 0.028. Prevalence of most relevant comorbidities and the Charlson comorbidity index were similar across the two groups. A high agreement with a correlation among both diagnostic methods was observed (Spearman's rho = 0.83, *P* < 0.001).

**Conclusion:**

Plasma GDF-15 displays promising prognostic properties for the prediction of long-term survival beyond clinical parameters in patients on maintenance haemodialysis.

## 1. Introduction

Patients with kidney failure requiring renal replacement therapy display a severe prognosis with high incidence of cardiovascular complications and death being several times higher than in the general population [1]. Accelerated vascular aging related to uraemia plays an important role. The final catastrophic clinical event is often preceded by a long phase of subclinical disease progression. Noninvasive identification of patients at high risk of cardiovascular events and death at an early stage might offer the opportunity to timely implement disease-modifying therapies and–hopefully–improve outcomes of these patients.

Circulating protein biomarkers might be helpful for this purpose. Natriuretic peptides and high-sensitive cardiac troponin have been shown to provide valuable prognostic information regarding cardiovascular events, but their value in patients on maintenance haemodialysis (HD) is impaired due to the peculiar metabolism with relevant renal clearance.

Novel biomarkers such as growth differentiation factor 15 (GDF-15), a distant member of the transforming growth factor beta superfamily, have been explored as a relevant gene in kidney injury [2] and as prognostic biomarker in multiple disease entities, including cardiovascular disease. GDF-15 is strongly upregulated in response to hypoxic, mechanical, oxidative, or inflammatory stress that contributes to disease progression. Today, GDF-15 is considered as a marker of integrated underlying cardiovascular disease burden with higher circulating levels being associated with the presence of atherosclerotic cardiovascular disease. Very recently, a large study across multiple populations with atherosclerotic cardiovascular disease showed that circulating GDF-15 is a robust biomarker for assessing the risk of cardiovascular events and death beyond established clinical factors and biomarkers [3].

The aim of this study was to assess the association between GDF-15 blood levels and mortality in a cohort of patients on maintenance HD.

## 2. Methods

The study was performed between October and December 2016 at the University Hospital of Zurich, Switzerland, with a clinical follow-up until January 2020 (up to 40 months). The study population consisted of 30 clinically stable chronic outpatients on maintenance HD using a FXCordDiax 100 filter (Fresenius Medical Care GmbH, Bad Homburg, Germany) in the haemodiafiltration mode. The original study was designed to analyse circulating plasma biomarkers at different levels of congestion and consisted of four visits with collection of clinical data and blood samples, as described previously [4].

Previous studies showed lower intraindividual and higher interindividual biological variation of GDF-15 [5, 6]. However, to overcome the impact of congestion on circulating GDF-15 in HD patients as previously shown by our group [7], for the purpose of this study, we measured GDF-15 only in samples from the first study visit and collected after a routine HD session. Blood samples were centrifuged within 6 h, and ethylene-diamine-tetraacetic acid (EDTA) plasma aliquots were stored at −80°C until further analysed. Circulating levels of GDF-15 were measured using the Proseek Multiplex cardiovascular disease (CVD) panels (Olink Proteomics AB, Uppsala, Sweden). The method is based on the proximity extension assay (PEA) technology, and the final assay readout is expressed in normalized protein expression (NPX) values, which is an arbitrary unit on a log2 scale with high values corresponding to high protein expression. Plasma concentrations of GDF-15 were validated and quantified on a Cobas®8000 analytical platform (Roche Diagnostics, Meylan, France) using the Elecsys GDF-15 electrochemiluminescence immunoassay on a Cobas E801 analyzer (Roche Diagnostics Meylan, France) in 26 of 30 subjects. The measuring range of the assay extended from 400 to 20,000 ng/L. Follow-up for survival was performed using the electronic medical record with direct contact by phone if needed. Data are presented as median (quartiles) or number (percentage), as appropriate. Group characteristics were compared with the Fisher's exact test or the Mann–Whitney *U* test, as appropriate. Survival was plotted with the Kaplan–Meier curve, and differences between groups were assessed by the log-rank test. The prognostic performance of GDF-15 was assessed by receiver operating characteristic (ROC) analysis and expressed as area under the ROC curve (AUROC). Correlation analysis was performed using Spearman's correlation coefficient (rho), and the agreement between the two different assays is plotted on the Bland–Altman plot. The study was performed according to the standards of the Declaration of Helsinki and approved by the local ethics committees of Zurich (2016-00451 and 2020-02087). All patients signed written informed consent forms. The study is registered in clinicaltrials.gov (NCT02962635). The null hypothesis was rejected with a two-sided*P* value <0.05. All analyses were performed with the use of IBM SPSS Statistics, Version 28.0 (IBM Corp, Armonk, NY, USA).

## 3. Results

Baseline characteristics have been previously published [4]. Briefly, the study population consisted of 30 patients on maintenance HD, predominantly middle-aged men with high burden of cardiovascular risk factors and established cardiovascular disease (Supplementary Table 1). Dialysis access was mostly an arteriovenous fistula, median dialysis vintage was 25 months, and anuria was present in about half of the patients. The median circulating GDF-15 levels were 5.3 (5.1–5.7) NPX resp. 2617 (1782–3702) ng/L.

**Figure 1:**
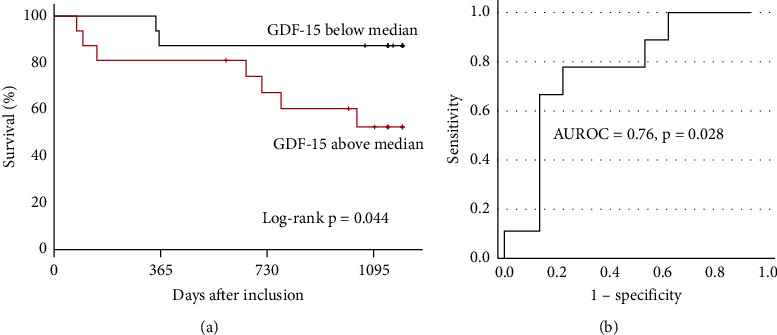
Prognostic properties of GDF-15 for 1-year mortality in haemodialysis patients. (a) Survival of haemodialysis patients according to circulating levels of GDF-15 after HD session. (b) Receiver operating characteristics (ROC) curve of GDF-15 for predicting 1-year mortality. AUROC: area under the receiver operating characteristic curve.

During a median follow-up of 38 months (23–40 months), 9 patients (30%) died. Seven deaths occurred in the group of patients with a circulating GDF-15 above the median and two in the group with lower GDF-15. The Kaplan–Meier diagram (Figure 1(a)) shows the higher mortality of patients with circulating GDF-15 levels above the median (red) compared to those with GDF-15 below the median, log-rank*P* = 0.044. The performance of circulating GDF-15 to predict long-term mortality is shown in Figure 1(b), with a AUROC of 0.76, *P* = 0.028. ROC curve analysis showed that a GDF-15 threshold of 1800 ng/L had a sensitivity of 100% and a specificity of 33% for predicting 1-year mortality. The optimal cut-off was at 2820 ng/L, which had a sensitivity of 78% and a specificity of 76% for predicting 1-year mortality.

Patients with higher circulating GDF-15 were nonsignificantly older, had a lower body mass index (24 vs. 27 kg/m^2^, *P* = 0.04), and a higher prevalence of peripheral artery disease (33% vs. 0%, *P* = 0.04) compared to patients with lower circulating GDF-15. The prevalence of other relevant comorbidities such as coronary artery disease, heart failure, atrial fibrillation, neoplasia, and the Charlson comorbidity index was not significantly different across the two groups. Furthermore, the clinical presentation at the index HD session was similar except for a significantly higher amount of overhydration (1.6 vs. 0 L, *P* = 0.05) and a higher prevalence of relevant overhydration (OH/ECV) (40% vs. 7%), in those with GDF-15 levels above the median, Table 1.

**Figure 2:**
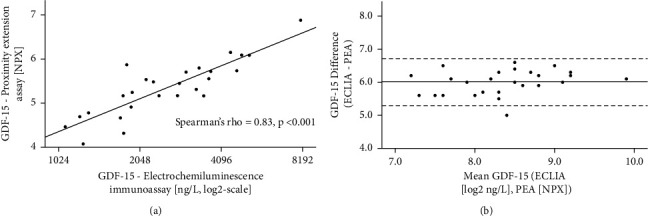
Correlation of GDF-15 values measured with the two methods. (a) Correlation of GDF-15 concentrations measured with electrochemiluminescent immunoassay (ECLIA) and proximity extension assay (PEA). PEA values are expressed in NPX (normalized protein expression), an arbitrary unit on log2 scale, ECLIA values in ng/L on a log2 scale. (b) Bland–Altman plot showing the agreement between the two methods. PEA values are expressed in NPX, and CMIA values have been log2-transformed. The continuous line shows the mean difference between the two assays, the dotted lines 1.96x standard deviation.

Finally, we confirmed the accuracy of the GDF-15 measurements with the proximity extension essay (PEA) on a standard electrochemiluminescence immunoassay (ECLIA). As shown in Figure 2(a), there was a very strong correlation between the two methods (Spearman's rho = 0.83, *P* < 0.001) with high agreement between the two different assays as plotted on the Bland–Altman plot (Figure 2(b)).

## 4. Discussion

Patients with kidney failure on maintenance HD are at particularly high risk of death, mostly of cardiovascular/atherosclerotic origin. Despite careful history and routine clinical assessment, the individual risk of death is difficult to ascertain due to atypical clinical manifestation of cardiovascular disease in HD patients and concomitant comorbidities. Circulating biomarkers can be used to facilitate prognostication and guide interventions potentially reducing the risk of adverse outcomes of patients at high risk. GDF-15 is a member of the transforming growth factor beta superfamily that is weakly expressed in most organs under healthy conditions. In human disease, GDF-15 is strongly upregulated in response to hypoxic, mechanical, oxidative, or inflammatory stress [8].

Given its properties, GDF-15 has been tested as a prognostic marker in multiple diseases, including coronary heart disease, heart failure, atrial fibrillation, and cancer. In atherosclerotic cardiovascular disease, GDF-15 has emerged as a marker of integrated underlying comorbidity burden beyond clinical factors with higher circulating levels of GDF-15 being associated with detrimental prognosis [8].

Our study, performed in a well-characterized cohort of patients on maintenance HD and with a follow-up of >3 years, could confirm the strong association between circulating GDF-15 and overall mortality. Indeed, patients with high GDF-15 displayed a 3-fold mortality compared to patients with lower GDF-15. Both groups were similar in respect to clinical presentation, cardiovascular disease (except for peripheral artery disease), and comorbidity burden (Charlson comorbidity index). Hence, circulating GDF-15 seems to capture the severity of comorbidities and predict long-term survival in a more comprehensive way beyond clinical history and established scores. Given the paucity if established biomarkers that robustly predict the risk of death in HD patients, our results suggest a potential role for GDF-15 to better predict long-term risk of death in HD patients.

Of note, a higher level of residual overhydration posthaemodialysis was observed in our patients with higher levels of GDF-15. Overhydration is a frequent condition and is associated with increased risk of death in HD patients and might arise either from renal salt and water retention and insufficient ultrafiltration during HD or concomitant congestive heart failure. In previous studies investigating mechanisms of fluid overload in HD, our group identified several circulating proteins that are upregulated in the presence of fluid overload. Among them, natriuretic peptides, vascular endothelial growth factor D (VEGFD), soluble CD146, and GDF-15 showed the strongest positive correlation with overhydration [4, 7]. In an attempt to elucidate why overhydration is associated with higher mortality of HD patients, in previous studies, we observed that the risk of death is particularly high in overhydrated patients with concomitant cardiovascular disease [4] and that overhydration *per se* has little impact on prognosis. Along this line, our current study shows that elevated GDF-15 is associated with overhydration and elevated risk of death possibly as a consequence of concomitant cardiovascular comorbidity burden.

Our data are in line with previous studies and with a recent large secondary patient-level analysis of eight randomized clinical trials across different forms of atherosclerotic cardiovascular disease, which observed a strong association between circulating GDF-15 and multiples types of cardiovascular events, including death and hospitalization for heart failure [3].

Compared to studies including healthy controls or patients with chronic atherosclerotic cardiovascular disease, patients with stable kidney failure on maintenance HD displayed markedly higher levels of circulating GDF-15. Indeed, median circulating GDF-15 in our cohort (∼2600 ng/L) was higher compared to patients with chronic coronary artery disease or atrial fibrillation (∼1000–2000 ng/L) or healthy controls (<1000 ng/L) but similar to patients with chronic heart failure (median ∼2000–3000 ng/L) [8]. This observation is of importance since it confirms the high comorbidity burden and similarities between kidney and heart failure, which both share similar triggers of GDF-15 and natriuretic peptide release such as myocardial stress, hemodynamic overload, and inflammation, among others.

Finally, our study confirmed the accuracy of the PEA technology for the quantification of GDF-15 compared with the standard ECLIA technique. These data are in line with previous studies for other biomarkers [9].

### 4.1. Limitations

Our study was performed in a small, well-characterized cohort, and generalizability needs to be validated in a larger, multicentric cohort. We report all-mortality because the precise cause of death could not be established for most of the patients. However, in light of the literature, a high proportion of cardiovascular causes of death can be anticipated. Furthermore, it is not known whether risk stratification by GDF-15 is useful in identifying groups of patients who will benefit from specific therapeutic interventions.

## 5. Conclusion

Our study describes promising prognostic properties of circulating GDF-15 to predict long-term survival beyond clinical parameters in patients on maintenance haemodialysis. Larger multicentric studies are needed to confirm these observations.

## Figures and Tables

**Table 1:** Baseline characteristics of the study population according to circulating GDF-15 after an HD session.

	GDF-15 below median	GDF-15 above median	*P* value
Age (years)	60 (54–76)	69 (61–82)	0.28
Male gender	12 (80%)	13 (87%)	1.00
Weight (kg)	77 (65–89)	71 (61–76)	0.10
Body mass index (kg/m^2^)	27 (25–32)	24 (22–27)	0.04
Coronary artery disease	6 (40%)	6 (40%)	1.00
Peripheral artery disease	0 (0%)	5 (33%)	0.04
Heart failure	3 (20%)	2 (13%)	1.00
Atrial fibrillation	3 (20%)	5 (33%)	0.68
Diabetes mellitus	6 (40%)	5 (33%)	1.00
Neoplasia	4 (27%)	8 (53%)	0.26
Charlson comorbidity index	6 (4–8)	7 (5–9)	0.14
Systolic blood pressure (mmHg)	129 (113–158)	132 (123–159)	0.62
Diastolic blood pressure (mmHg)	70 (61–79)	69 (63–81)	0.81
Heart rate (/min)	78 (68–89)	78 (69–86)	0.84
Overhydration after haemodialysis (L)	0 (−3.7–1.4)	1.6 (0.1–3)	0.05
OH/ECW > 0.15 after haemodialysis	1 (7%)	6 (40%)	0.06
Dialysis vintage (days)	868 (404–1319)	563 (189–1238)	0.59

Overhydration was quantitatively assessed with a portable whole-body bioimpedance spectroscopy device (Fresenius medical care GmbH, Bad Homburg, Germany). Relevant overhydration was previously defined as a quotient of overhydration (OH) over extracellular water (ECW) > 0.15 according to the literature.

## Data Availability

The data used to support the findings of the study are available from the corresponding author upon request and approval of the ethical committee.

## Supplementary Materials

Supplementary Materials Supplementary Table 1 summarizes the baseline characteristics of the study population.
