# Supplementary material for: Growth Differentiation Factor 15 and Risk of Death in Haemodialysis Patients
Source: Int J Nephrol. 2023 Mar 23;2023:5163548. doi: 10.1155/2023/5163548 (PMC10063357; doi:10.1155/2023/5163548)
Supplement: Supplementary Materials — Supplementary Table 1 summarizes the baseline characteristics of the study population. [file 5163548.f1.docx]

**Supplementary Table 1. Clinical parameters of the study cohorts (N=30)**

| Age (years) | 67 [56-78] |
| --- | --- |
| Gender (male) | 25 (83%) |
| Arterial hypertension | 30 (100%) |
| Diabetes | 11 (37%) |
| History of coronary artery disease | 12 (40%) |
| History of peripheral artery disease | 5 (17%) |
| History of chronic heart failure | 4 (13%) |
| Dialysis vintage (months) | 25 [12-42] |
| Dialysis access  AV fistula/graft  Catheter | 29 (97%)  1 (3%) |
| Anuria (< 500ml/d) | 14 (47%) |
| Number of antihypertensive drugs | 2 [1-3] |
| Number of diuretics | 1.5 [0-2] |

Data are presented as median and interquartile range or number and percentage as appropriate
